# Environmental Impact of Phosphogypsum-Derived Building Materials

**DOI:** 10.3390/ijerph17124248

**Published:** 2020-06-14

**Authors:** M. I. Romero-Hermida, V. Flores-Alés, S. J. Hurtado-Bermúdez, A. Santos, L. Esquivias

**Affiliations:** 1 Física de la Materia Condensada, Universidad de Sevilla, 41012 Sevilla, Spain; isaromerohermida@us.es (M.I.R.-H.); luisequivias@us.es (L.E.); 2 Construcciones Arquitectónicas II, Universidad de Sevilla, 41012 Sevilla, Spain; 3 CITIUS—S.G.I. Celestino Mutis, Universidad de Sevilla, 41012 Sevilla, Spain; shurtado@us.es; 4 De Ciencias de la Tierra, Universidad de Cádiz, 11519 Cádiz, Spain; alberto.santos@uca.es; 5 Instituto de Ciencia de Materiales de Sevilla, CSIC—Universidad de Sevilla, 41092 Sevilla, Spain

**Keywords:** phosphogypsum, lime paste, mortar, heavy metals, radionuclides

## Abstract

The aim of the present work was to characterize the products obtained from the treatment of phosphogypsum residue by means of two recovery routes, and also to evaluate the concentrations of heavy metals and radionuclides in the materials obtained and their leachates. In this way, it is possible to determine how the most hazardous components of phosphogypsum behave during procedures until their stabilization through CO_2_ fixation. This study provides an initial estimate of the possibilities of reusing the resulting products from a health and safety risk standpoint and their potential polluting capacity. The phases resulting from the transformations were controlled, and the behaviour of standard mortars manufactured from the resulting paste lime was studied. In all cases, an additional control of the leachate products was performed.

## 1. Introduction

One of the most contaminating industries is the phosphoric acid (H_3_PO_4_) industry. This chemical is widely used in agricultural fertilizers, detergent additives, cleaning products, and insecticides. Phosphoric acid is produced from the treatment of calcium phosphate rocks with sulphuric acid. The chemical reaction of the industrial process is:

Ca_3_(PO_4_) + 3H_2_SO_4_ + 6H_2_O → 2H_3_PO_4_ + 3(CaSO_4_)·2H_2_O. (1)

The residue generated by this process, that is, mainly calcium sulphate di-hydrate, is commonly known as phosphogypsum (PG) [1].

PG is normally slurred with water and then pumped in enormous amounts to a nearby deposit. The fertilizer manufacturing industry in the province of Huelva (SW Spain) is based on an important production of phosphoric acid by wet processing of the phosphoric rock in an industrial plant from the 1960s. In some cases, such as in Huelva (SW Spain), these deposits may be located in the vicinity of populated towns or in coastal zones close to the phosphoric acid plants where they occupy large areas of land, thus representing a hazard to both the environment and local population [2,3,4]. In the case of Huelva (SW Spain), the material stored reaches 120 million tons and occupies an area of 1200 hectares near the estuary of the Tinto and Odiel river mouths [5]. The growing interest in the restoration of the environment by removing landfills and waste stacks is an incentive to search for potential low-cost applications of PG wastes.

Untreated PG could certainly be used in several industrial applications. However, it contains heavy metals and radionuclides, which are harmful to the environment and human health [6,7]. This is an important drawback when it comes to reincorporating PG in the industrial production circuit in accordance with the principles of the clean circular economy [8]. Each year, nearly 200 Mt of PG are produced worldwide, but only 15% of pristine PG is re-used [9,10]. The properties of the material not only make it urgent to process and eventually remove the deposits entirely, but also offer many significant opportunities to recover valuable materials, thus supporting the circular economy and, as mentioned, adding value by CO_2_ sequestration.

The incorporation of construction and demolition waste in concrete and cement mortars is already a common practice, and the aim is to ensure the construction industry assumes part of the waste it produces [11,12]. The goal of this stream is to promote the use of conglomerates by incorporating waste to produce so-called “green concrete”. To achieve this, appropriate standards are urgently required, as well as interdisciplinary collaborations between the different stakeholders involved in construction [13].

PG is a source of calcium, which, in addition to being a CO_2_ sequestering agent, is a construction material [14,15]. Research has demonstrated the high efficiency of portlandite precipitation by PG dissolution using an alkaline soda solution [16]; PG, reacting with ammonia, may be converted into ammonium sulphate and precipitated calcium carbonate [17], yielding reaction conversion efficiencies of >95% [18]. By means of other processes with PG, wastes, which are unable to fix CO_2_ by themselves, can be successfully turned into effective CO_2_ sinks [19]. CO_2_ sequestration may also be achieved through mineral carbonation of waste PG using the technique of membrane electrolysis [20] and by the PG reduction thermal decomposition process [21].

In this study, PG was treated in two ways. The first option (procedure A) was based on its reaction with sodium hydroxide (Na(OH)) to obtain portlandite (Ca(OH)_2_) in the form of lime paste in a thenardite (Na_2_SO_4_) solution [20]. Then, the lime reacts rapidly and completely with CO_2,_ yielding precipitated calcium carbonate (PCC). The second option (procedure B) was based on dissolution in a highly alkaline liquid residue from the aluminium anodizing industry, rich in Na-Al. The resulting precipitate consisted of katoite (Ca_3_Al_2_(OH)_12_). Katoite has high carbonation efficiency (80–100%), and produces a solid precipitate mainly composed of calcite (CaCO_3_) and an aluminium hydroxide (Al(OH)_3_) solution [22].

Following the stream to reuse these wastes in the construction industry, the slaked lime produced can be used as a mortar component. Ca(OH)_2_ improves the degree of cohesion of the materials by means of the carbonation reaction, thus reducing porosity. Its carbonation is a spontaneous process driven by diffusion and affected by natural variables [23].

PG is classified, according to the United States Environmental Protection Agency (US-EPA), 2018 [24], as a Technologically Enhanced Naturally Occurring Radioactive Material (TENORM). The main toxic and radioactive metals present in PG are Sr, As, Y, Cu, Pb, and the radioactive isotopes ^226^Ra, ^238^U, ^230^Th, ^210^Pb, and ^210^Po [25,26,27]. Minority impurities, such as P, Cd, and Cr are also dragged out. These represent a risk of environmental contamination that affects living beings. The concentrations of these elements vary depending on the characteristics of the original phosphate rock [28]. 

To establish the limitations of the use of PG related to its content in the abovementioned elements, it is necessary to understand their leaching processes during treatment [29], from PG until its final stabilization as calcium carbonate. Thus, the environmental impact generated by its extraction from PG rafts and its possible recycling could be evaluated.

The main aim of this work is the characterization and control of the phases resulting from PG waste transformation by two processes: Procedure A is based on treatment with Na(OH) in which the controls were performed on the portlandite obtained as lime paste in the first phase and also in the calcite resulting from carbonation. Additionally, lime mortars were manufactured from lime paste and standardized siliceous sand to verify the behaviour once the resulting material was stabilized [30], as well as its possible viability as construction material. Procedure B is based on the reaction with the residual liquid of the aluminium industry. It was carried out on the katoite from the first reaction and the final calcite obtained after the carbonation process.

In all cases, an additional control of the leachate products in accordance with the toxicity characteristic leaching procedure (TCLP) was carried out [31].

## 2. Materials and Methods

**Figure 1:**
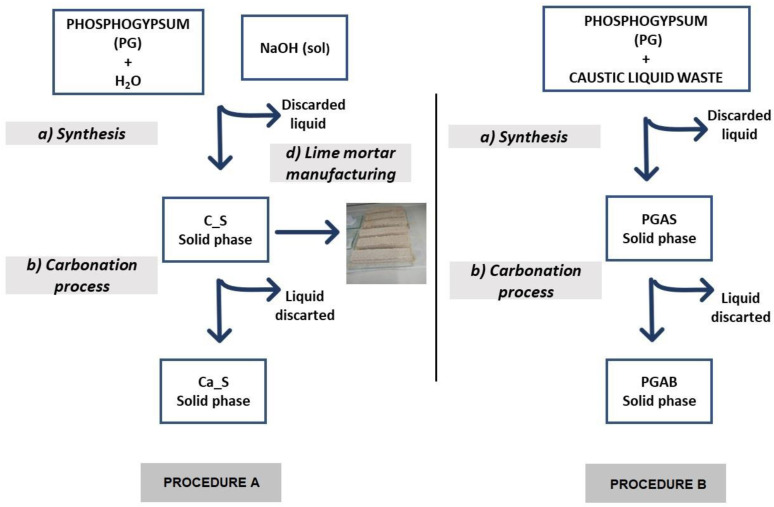
Schemes of both chemical processes.

Crude solid PG was supplied by Fertiberia from the stacks in Huelva, Spain. For its characterization, the residue was homogenized by means of a splitter. Then, the moisture content of raw PG was analysed, yielding a value of 21%. For the different tests, the remaining moisture was removed in an oven at 40 °C for around 48 hours to preserve the structural water of the gypsum. Once dry, it was grounded in a mortar and used in the experiments without any other treatment. The process schemes can be seen in Figure 1.

### 2.1. Procedure A

#### 2.1.1. Synthesis

Lime putty was obtained by using the Cárdenas–Escudero method [16], with some modifications to scale the generation of by-products.

In the first stage, 300 g of PG was suspended in 500 mL of distilled H_2_O under magnetic stirring. To this suspension, 180 g of NaOH in a 9 M solution was slowly added to favour the crystallization of the solid phase. The mixture was also stirred for the next 10 min. The reaction associated with this process was:

CaSO_4_·2H_2_O + 2NaOH → Ca(OH)_2_ + Na_2_SO_4_ + 2H_2_O. (2)

This process resulted in the precipitation of a whitish solid phase identified as putty lime and the Na_2_SO_4_ solution as a clear supernatant liquid. The solid phase was separated by centrifugation and labelled C_S. The liquid phase was discarded for this research. Several samples of the solid phase were dried in an oven at 40 °C in order to hydrate and carbonate them to preserve them from humidity and atmospheric CO_2_, so that they could be studied.

#### 2.1.2. Carbonation Process

In the second stage, 2 g of the C_S sample was dispersed in 40 mL of distilled water under magnetic stirring in a reactor (mass ratio [C_S]/[H_2_O] = 1/20). A flow of CO_2_ (1 bar, 20 cm^3^/s) was bubbled through the suspension for 15 min at room temperature and pressure [1]. The sample was left to rest overnight in this CO_2_-rich water.

The carbonation reaction was:

Ca(OH)_2_ + CO_2_ → CaCO_3_ + H_2_O. (3)

The resulting solid phase, labelled Ca_S (mainly CaCO_3_,), was separated by centrifugation and dried in an oven at 80 °C. The supernatant was discarded, and samples of the solid phase were taken for study.

#### 2.1.3. Lime Mortar Manufacturing

Mortars were manufactured with lime putty obtained as described above, with 1:3 lime/sand and 0.5 by weight water/lime ratios. They were prepared with a water content that allowed a consistency of 185 mm, measured in accordance with the UNE-EN 1015-3/A1 standard [32] to ensure its workability. 

Prismatic samples were prepared in accordance with UNE-EN 1015-2/A1 standards [33]. The samples were then deposited for 21 days in climatic chambers (New Brunsaick Galaxy 170) to accelerate carbonation under realistic curing conditions of 25 °C, 50–60% RH, and 10% vol. of CO_2_ concentration.

### 2.2. Procedure B

#### 2.2.1. Synthesis

Verinsur S.A. (Jerez de la Frontera, Spain) provided the aluminium anodizing process waste. This was a clear solution with a pH of around 14, containing a small amount of a precipitate phase. For this study, the residue was filtered, and the precipitate discarded. The caustic liquid waste had a density of 1.32 g/cm^3^, and a composition of [Na] = 110 ± 2 g/L (4.78 ± 0.09 M)) and [Al] = 52 ±2 g/L (1.93 ± 0.07 M).

The caustic liquid from the anodizing aluminium industry reacted with PG according to the reaction [23]:

3(CaSO_4_·2H_2_ O) + 6Na^+^_(aq)_ + 12OH^-^_(aq)_ + 2Al^3+^_(aq)_ ↔  3Na_2_SO_4_ + Ca_3_Al_2_(OH)_12_ + 6H_2_O. (4)

The process was initiated by adding 12.5 g of PG to 25.0 mL of the aluminium-anodizing residue under magnetic stirring for 3 h in ambient pressure and temperature conditions [9,16]. The pH was 12.0 during the process. The formation of a grey precipitate was observed, labelled as PGAS, and a yellowish supernatant was discarded for this study. Both phases were separated by centrifugation, and then the solid phase was placed in an oven at 80 °C to preserve it from hydration and carbonation. 

Based on the chemical composition of the residues, this specific mass ratio corresponded to a stoichiometric molar ratio of [Ca^2+^]/[Al^3+^] = 1.5, targeting the chemical reaction (4). It should be noted that other stoichiometric relationships with different additions of water were tested. In this study, only the one that produced the best CO_2_ sequestering results, which was the final purpose, is presented.

#### 2.2.2. Carbonation Process

In this step, 2 g from the PGAS sample were dispersed in 40 mL of distilled water [19]. The obtained mixture was subjected to a continuous flow of pure CO_2_ (≈1 bar, 20 cm^3^/s) under magnetic stirring. The sample was carbonated according to the following reaction:

Ca_3_Al_2_(OH)_12_ + 3CO_2_ → 3CaCO_3_ + 2Al(OH)_3_ + 3H_2_O. (5)

The pH during the process decreased monotonously from 12.8 until it stabilized at 6.7 after 110 min. This resulted in a new solid–liquid suspension whose phases were separated by centrifugation. The solid phase was labelled PGAB, and the liquid phase discarded. The reproducibility of the experiment was verified several times. 

### 2.3. Characterization Techniques

Mineral characterization of the samples was performed by X-ray diffraction. The diffraction intensities were measured on a Bruker powder diffractometer (model D8-Advance A25) equipped with conventional Bragg–Brentano geometry and a Cu anode. X’Pert HighScore software (Malvern Panalytical, Malvern, UK) was used to analyse the results.

An ICP-MS/MS Agilent 8800 (Agilent Technologies, Santa Clara, CA, USA) was used to analyse the elements and isotopes at trace and ultra-trace levels (ppm–ppt ranges). This equipment was provided with an octopolar reaction system (ORS), designed to minimize different types of spectrometric interference. The elementary concentrations in liquid samples were analysed with a conventional nebulizer Savillex X400 (ISC-Science. Oviedo, Spain) coupled with a CETAC ASX 520 sample introduction system. A specific interface allowed the samples to be analysed in the presence of high concentrations of hydrofluoric acid. Quantitative analyses were performed in accordance with the US-EPA 200.8 (1986) [34]. The solid samples were subjected to microwave-assisted acid digestion in 9 mL of concentrated HNO_3_ and 3 mL of HF for 15 min at 200 °C. After cooling, the contents of the vessel were filtered and transferred to a 25 mL volumetric flask with type I water from a Milli-Q Integral-3 (Millipore, Merck, Spain).

The activity concentrations of the natural radionuclides were measured by high-resolution gamma-ray spectrometry. The detection system used consisted of a Reverse-Electrode Germanium (REGe) detector, model GR6040 (Canberra. Montigny-le-Bretonneux, France) shielded by an active anti-coincidence system containing an annular anti-Compton NaI detector. Canberra GENIE 2000 (Canberra. Montigny-le-Bretonneux, France) software was used to obtain spectra and for subsequent analysis. Canberra LABSOCS software (Canberra. Montigny-le-Bretonneux, France) was used to calculate the counting efficiency of the germanium detector. Cylindrical containers (liquid samples) and Petri dishes (solid samples) were used in the experimental measurements and sealed under vacuum to prevent the escape of radon gas, enabling achievement of secular equilibrium between radon and its daughters (^226^Ra and ^214^Pb). The activity concentrations of the gamma emitters were determined through the following energies: ^210^Pb (46.5 keV), ^232^Th (63.3 keV), ^226^Ra (351 keV of ^214^Pb), ^235^U (144 keV), and 40 K (1460 keV).

The TCLP (1311 US-EPA) leaching test was performed to assess the effectiveness of the immobilization of the different components and obtain the degree of toxicity associated with the different residues. This procedure used an extraction liquid of pH 2.88 ± 0.05 consisting of 5.7 mL of glacial acetic acid diluted with 1L with deionized water. Of the solid sample, 100 g was added to an amount of extraction liquid, maintaining the 20 mL/g ratio, and placed in a rotary system at 30 ± 2 rpm for 18 hours and at a temperature of 22 ± 3 °C.

A Rh-tube Panalytical X-ray Fluorescence Spectrometer (AXIOS model) (Malvern Panalytical. Malvern, UK) was used to apply the XRF technique, enabling qualitative and quantitative chemical analysis from O to U in a wide range of concentrations, from major components to traces.

## 3. Results and Discussion

### 3.1. Evolution of Environmental Risk

#### 3.1.1. Evaluation of Major Elements and Trace Elements from the Treatment of PG with a Soda in Solution

(a) Phosphogypsum

The XRD analysis confirmed the almost exclusive presence of gypsum in the sample of unprocessed PG [17], with a residual amount of quartz (SiO_2_) originating from the mother phosphate rock.

According to the XRF analysis results, shown in Table 1, the PG was mainly composed of Ca (32 wt. %, as CaO) and S (46 wt. %, as SO_3_). These results were similar to those reported in other studies [1] and corresponded to a Ca/S ≈0.993 molar ratio, very close to the expected Ca/S ≈1 molar ratio. The main impurities of the PG were Si (2.52 wt. %, as SiO_2_) and P (0.65 wt. %, as P_2_O_5_).

The main heavy metals and radionuclides observed in the PG were: Sr, Cr, As, Cu, Cd, Pb, U, and Th. The minority elements identified are shown in Table 2, together with the PG sample leaching results obtained from TCLP.

The concentrations of V, Cr, Ba, Pb, and Th were well below those typically found in undisturbed soils (Table 2) [35]. However, the concentration of Cd was ≈20 times higher than the typical value, the concentration of Sr was ≈1.2 times higher, and the content of U was approximately half of its value in uncontaminated soils. It should be noted that the concentrations of Cd and Sr were lower than different PG sources analysed in the literature (Idaho, South Africa, and Tunisia) [29]. The concentrations of other metals were below the detection limit.

All these results, when compared with the limit values permitted by the US-EPA for metals in leachates extracted with the TCLP test (Table 2), were well below the established limits. No information is available for some metals, such as V, Co, Ni, Zn, and Sr. More strict criteria would be applicable if the aforementioned numbers are compared with those stipulated in legislation regulating drinking water for human consumption. For this reason, the maximum permissible limits of the metals studied were taken in accordance with the US-EPA (1986), the World Health Organization, and the EU (Drinking Water Directive, 1998) (Table 2) [36]. Additionally, for vanadium, German legislation was taken as a basis for purification [37,38]. Even so, the level of leachate concentration did not exceed the established limits, except for Ni, which slightly exceeded these limits (30%).

Considering the results obtained, the PG analysed in this study does not generate major environmental risks, and therefore, no corrective measures should be applied. However, caution must be taken before any categorical assertion, because PG is heterogeneous, and its heavy metal and radionuclide contents depend on the depth at which the PG is found [39,40,41].

(b) Soda solution treatment by-products

**Figure 2:**
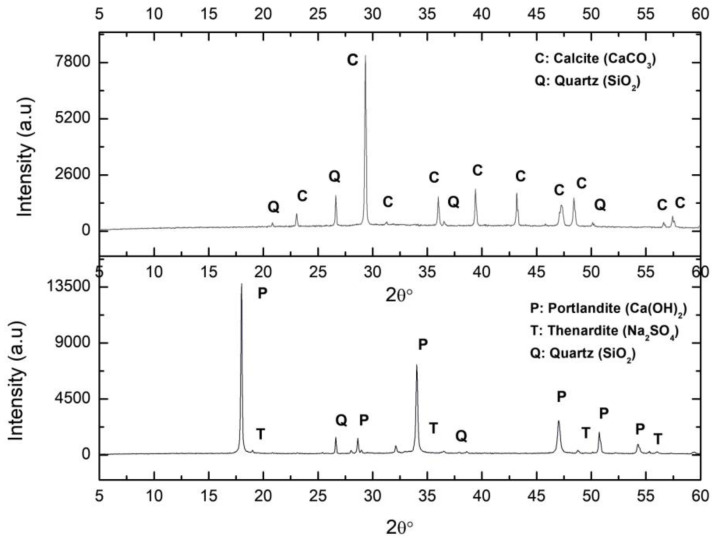
XRD (X Ray Diffraction of the samples Ca_S (calcite from lime putty carbonation) (above) and C_S (lime putty) (below). C: Calcite, Q: Quartz, P: Portlandite, T: Thenardite.

The XRD diffractogram of the C_S sample is shown in Figure 2. As expected according to its composition (Table 1), there was a major presence of CaO, corresponding to slaked lime and Na_2_O and SO_3_ due to the sodium sulphate. The lime was composed of 65.3% Ca(OH)_2_, 13.5% SO_3_, silica impurities (quartz), and phosphates (1.02% P_2_O_5_), and did not contain MgO.

The UNE-EN 459-2 standard [42] states that lime content must always be higher than 55%, although higher values may be required. Nevertheless, the UNE-EN 196-2: 2014 standard [43] requires that sulphate content, given in terms of SO_3_, must be less than 2%. Regarding the MgO content, the standard requires this to be less than 5%. The SO_3_ content exceeded the limit established in the regulations, and therefore, preventive measures are required to correct it.

The concentrations of Cr, Co, Ni, Zn, and Th (Table 3) were below the average concentrations found in typical uncontaminated soils. Those of As and Sr were higher by factors of ≈1.5 and 1.3, respectively. However, the concentrations of Se, Cd, U were substantially higher, as much as ≈37.5 times higher in the case of Cd. The increase in the concentration of U, ≈4 times higher than that of typical soil, was not surprising since it is a material obtained from PG, which is considered a NORM material, that is, one which is rich in U-Th radionucleide series.

(c) Carbonation by-products

The XRD analysis of this sample indicated that the portlandite carbonated completely, resulting only in calcite (Figure 2). The characteristics of this sample have been reported elsewhere [1].

In this case (Table 3), as may be expected, the concentrations of trace elements and radionuclides coincided almost completely with those found in the C_S sample.

(d) Mortar probes

**Figure 3:**
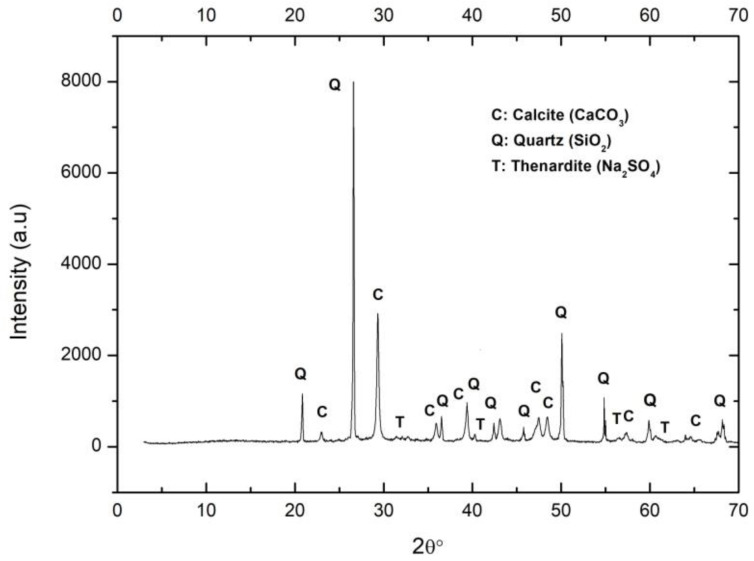
XRD of mortar sample. C: Calcite, Q: Quartz, T: Thenardite.

As expected, the majority in the discarded sand was calcite. The portlandite was fully carbonated. Small reflections of residual thenardite in the lime were also observed (Figure 3).

In the case of the mortar, since it is a very heterogeneous system on a millimeter scale, it was necessary to take different aliquots to obtain reliable information because the masses that can be digested by ICP are very small, resulting in a dispersion of the concentrations of the trace elements. The average results of four aliquots taken are presented in Table 3. It can be observed that, when mixing the lime with sand, the initial concentrations of most of the trace elements decreased. The average concentrations of As and Sr were below the limits established by current regulations. However, those of Se, Cd, and U were moderately above those limits. Besides the trace element concentrations, the results from the application of the TCLP technique are shown for the L_p, L_c, and L_m samples in Table 3. These results are compared with the maximum permissible limits established by the TCLP (US-EPA), indicated in Table 2. It has been verified that all the values were well below the established limits. Thus, it can be concluded that the monitored metals were effectively immobilized. It was also verified that these values were below the permissible limits established by TCLP (US-EPA). However, there is no information on permissible limits for some metals, such as Zn, Ni, V, and Sr. The concentrations of all the elements screened did not exceed the maximum permissible limits for drinking water. The V limit exceeded the maximum permitted by German law by a factor of ≈2.2.

#### 3.1.2. Contents of Major and Trace Elements in the Treatment of PG with Aluminium Residue

(a) Aluminium waste

The density of the aluminium residue was 1.32 g/cm^3^ measured by Hg pycnometry with pH = 14 and [Na] = 110 ± 2 g/L (4.78 ± 0.09 M) and [Al] = 52 ± 2 g/L (1.93 ± 0.07 M), according to ICP-OES results.

(b) Synthesis

**Figure 4:**
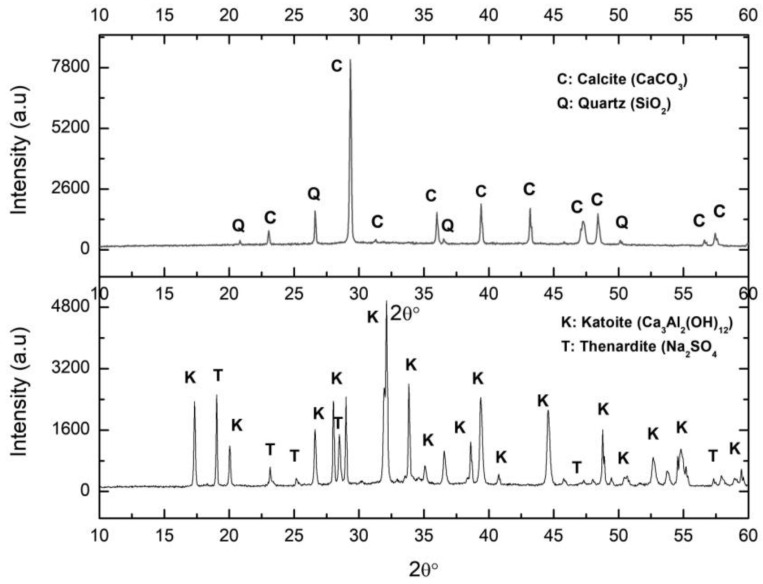
XRD of the samples PGAB: calcite (above) and PGAS: katoite precipitate (below). C: Calcite, Q: Quartz, K: Katoite, T: Thenardite.

The XRD pattern of the solid by-product resulting from an attack of the PG with the aluminium waste is represented in Figure 4, labelled PGAS. The main associated reflections corresponded to katoite and thenardite, as expected according to Equation (3). It should be noted that no residual gypsum was detected, indicating that the reaction was complete. The compositional analysis of the PGAS by-product by XRF (Table 1) confirmed the presence of Ca, Al, S, and Na as the main components, in the relative molar fractions Ca:Al (1.51) and Na:S (1.96), typical of katoite and thenardite, respectively, confirming that the reaction was indeed complete.

(c) Carbonation.

The XRD pattern of the carbonated sample (Figure 4) presents the calcite reflections accompanied by those of the remaining quartz impurities from the PG. No thenardite reflections were observed, confirming its complete dissolution during the carbonation process. However, the XRF analysis (Table 1) revealed the presence of substantial contents of certain chemical species not detected by XRD, indicating their amorphous character. These were mainly Al and S, and to a lesser extent, Na.

The contents of trace elements present in the PGAS sample (Table 4), such as V, Cr, As, Sr, Ba, and Th were well below the concentrations in undisturbed soils. However, the concentration of Cd (1.1 ± 0.2 mg kg) exceeded the typical concentration by a factor of 12.5. In contrast, the concentration of U (2.5 ± 0.3 mg/kg) was within the limit permitted for uncontaminated soils. 

In the case of the PGAB sample, the concentrations of the trace elements were well below the allowed concentrations for undisturbed soils, except for Cd (1.6 ± 0.2 mg/kg), which exceeded it by a factor of 17.5. In contrast, the concentration of U (2.9 ± 0.2 mg/kg) was moderately higher than the limit value.

The results of the TCLP leaching test in the PGAS and PGAB samples (whose liquids are labelled (L_k) and (L_ck), respectively) are shown in Table 4. All the resulting values for the monitored metals, compared with those in Table 2, were well below the permitted limits; hence, it may be concluded that these metals were effectively immobilized. As in the previous case, due to the lack of data on the maximum permitted limits of Zn, Ni, V, and Sr, reference was made to the limits established in the legislation that regulates water for human consumption. Values above those allowed were not observed.

### 3.2. Radiological Evolution

#### 3.2.1. Procedure A

Table 5 shows the concentrations of radionuclides obtained by gamma spectroscopy. ^226^Ra concentrations of PG samples coincide with results published elsewhere [1,4,26]. Similar conclusions were also obtained for ^232^Th and ^210^Po. In contrast, the concentrations of U isotopes were much lower than the rest of the radionuclides in the series. However, ^210^Pb presented very high activity due to the high solubility of U in acidic media; more than 85% of the phosphate rock content remains in the phosphoric acid fraction obtained during the industrial process, while on the contrary, more than 90% of the ^226^Ra and ^210^Pb ended up in the PG [44,45]. Finally, the Th series radionuclides presented very low levels in the PG sample, even lower than the concentrations in typical undisturbed soils (25–40 Bq/kg). These PG samples therefore contained concentrations below 1000 Bq/kg for U-series radionuclides (^226^Ra, ^210^Pb) and ^232^Th. Therefore, for this series, PG is not considered a NORM material by the IAEA regulation (2004) [46]. However, the high concentration of the ^226^Ra isotope makes it a NORM material, being the most important source of radioactivity in PG. The ^226^Ra produces radon gas (^222^Rn), which has a short half-life (3.8 days), an intense radiation capacity, and causes significant damage to internal organs (US-EPA, 2002) [47]. 

In the U.S., to prevent environmental and public health risks, the commercial use of PG for agriculture is limited for certain applications, such as soil amendment, and is only permitted if the average certified concentration of ^226^Ra does not exceed 370 Bq/kg (US-EPA, 1992) [48]. However, it is more important to know the concentration of radioisotopes when leaching occurs, rather than the absolute amount of radionuclides that has contributed to the soil. Table 3 shows the results of the leaching test using TCLP. As no information is available in this regard, the results were compared to the radioactivity levels in water for human consumption permitted by the US-EPA (2000) [49] and in Spanish regulations (Royal Decree, 2016) [50]. Some concentrations of radionuclides were below the measurement detection limits. In these cases, these values were taken as the upper limit of unfavourable conditions. For the PG sample, the results indicated that the total alpha and beta activities (0.65 Bq/L and 4 Bq/L, respectively) exceeded the radioactivity permitted for human drinking water according to the US-EPA and, obviously, the Spanish legislation (0.1 Bq/L and 1 Bq /L for alpha and beta activities, respectively). If the limit thresholds are exceeded, the specific radionuclides must be analysed and the correct measurements must be obtained. In PG leachate, concentrations of ^226^Ra and ^210^Pb exceeded the reference levels, although both radionuclides had relatively small transfer factors (Table 5), indicating that most of these radionuclides remained in the starting material; these results are consistent with values reported elsewhere [51,52].

C_S and Ca_S in Table 5 are the concentrations of radionuclides obtained in the lime and calcite, respectively, generated by the treatment of PG with caustic soda. These results confirmed that the major parts of the radionuclides accumulated in the Ca containing by-products [1,30]. The concentrations in the C_S samples of the uranium series exceeded the value established by CSN (UNSCEAR, 1993) at 1000 Bq/kg [53]; therefore, both by-products were considered NORM materials and must therefore be radiologically controlled for commercial applications.

The results of the TCLP test in the C_S sample presented with higher levels of total alpha and beta activities, 2 Bq/L and 3 Bq/L respectively. These values exceeded those allowed in water for human consumption. The ^226^Ra and ^210^Pb values must be below 0.5 Bq/L and 0.2 Bq/L, respectively, but both values were lower than their detection limits, being higher than the maximum permitted values. In the case of Ca_S, both total alpha and beta activity indexes (1.55 Bq/L and 2.15 Bq/L, respectively) also exceeded the permitted limits in water for human consumption. The leaching results of ^226^Ra and ^210^Pb in calcite exceeded the reference values, albeit with very small transfer factors, indicating that these radionuclides mainly remain in starting material.

Most of the radionuclides present in the mortar samples belong to the uranium series (Table 5). Neither ^226^Ra nor ^210^Pb exceeded the values established at 1000 Bq/kq and 5000 Bq/kg, respectively; therefore, mortar is not a NORM material. However, the values obtained with the TCLP test for total alpha and beta activities (1.85 Bq/L and 2 Bq/L, respectively) exceeded those permitted in water for human consumption. It should also be noted that their transfer factors were quite small.

#### 3.2.2. Procedure B

Table 5 also shows concentrations of radionuclides in katoite (PGAS) and calcite resulting from its carbonation (PGAB). In general, most of the radionuclides present in PG accumulate in solid by-products [23]. The concentrations of uranium series (^226^Ra and ^210^Pb) in PGAB and PGAS did not exceed the values established at 1000 Bq/kq and 5000 Bq/kg, respectively. Consequently, these by-products are not NORM materials and no radiological control for commercial applications is required.

In both cases, the total alpha and beta activity obtained with the TCLP test exceeded the permitted levels in water for human consumption. Specifically, the ^226^Ra content exceeded the reference value. The transfer factor of ^226^Ra for katoite was low but not so low for calcite, particularly when compared to that of calcite obtained by treating PG with reactive grade soda.

### 3.3. Radiological Risk Indexes

The purpose of measuring radioactive concentrations is to determine the risk of radiation emitted by these materials establishing so-called "radiological risk indexes". Two of the most widely used indexes are the "gamma activity concentration index" and “radium equivalent activity index”. 

The gamma activity concentration index (I_γ_) [53,54] is the sum of the contributions of the different natural radionuclides to the external gamma dose defined by the European Commission (EC-Radiation Protection, 1999) [55]:

(6) $$I_{\gamma }\equiv \frac{C_{\mathrm{Ra}226}}{300}+\frac{C_{\mathrm{Th}232}}{200}+\frac{C_{K40}}{3000},$$

where the I_γ_ index is correlated with the annual dose rate due to excess external gamma radiation caused by surface materials. The value established for no-risk materials is I_γ_ ≤ 1. Index values I_γ_ ≤ 2 correspond to a dose rate criterion of 0.3 mSv/year, while 2 < I_γ_ ≤ 6 corresponds to a criterion of 1 mSv/year (Miró et al., 2010). EU legislation aims to prevent the general population from receiving effective dose rates that are above 1 mSv/year with respect to the natural background, which corresponds to I_γ_ > 6. 

Radium equivalent activity, Ra_eq_, was used to compare the specific activity of materials containing different amounts of ^40^K, ^226^Ra, and ^232^Th. Ra_eq_ was calculated, in Bq/kg, from the following relationship [56,57,58]:

Ra_eq_ = (C_Th_ 1.43 + C_Ra_ + C_K_ 0.077) ≤ 370 Bq/kg, (7)

with C_Th_, C_Ra_, and C_K_ being specific activities of ^232^Th, ^226^Ra, and ^40^K, respectively (Bq/kg), and representing the risks of external gamma radiation. 

When the concentration of any radionuclide is below the detection limit, this value is taken for calculations. This provides upper limits for these indexes, corresponding to the most unfavourable scenario.

For PG, an index equal to 2 was obtained, and it could be used to produce a surface material with restricted use in construction, as it fulfils I_γ_ ≤ 6 (UNSCEAR, 1993) [50]. Although this material cannot be used without any restriction from the radiological standpoint, it is competitive when compared with thionite or untouched sludge from ilmenite, a NORM residue generated in the industrial process used to obtain titanium dioxide, whose index is I_γ_ = 16.7, or fly ash with I_γ_ = 3.90 [59]. 

An equivalent radioactivity of 370 Bq/kg in construction materials is assumed to produce an exposure of around 1.5 mGy/year in the population [60], a value considered to be the maximum limit by the ICRP (1977) [61]. 

The value of Ra_eq_ for PG was 580 Bq/kg, exceeding the established maximum value for construction materials. Thus, for the use of this waste as construction material, a radiological study is required for each specific application. 

The by-products from treatment of PG with caustic soda, C_S and Ca_S, presented I_γ_ of 2.4 and 2.7, respectively, and Ra_eq_ of 710Bq/kg and 800 Bq/kg, respectively, exceeding both values established in current regulations, which is as expected since the radionuclides in PG, ^226^Ra, ^238^U, ^230^Th, and ^210^Pb were almost completely transferred first to portlandite and then to calcium carbonate. The observed increase in radioactivity has to take into account mass loss after discounting the mass of sulphate ions of thenardite, which is free of radionuclides. However, both by-products could be used to produce surface material with restricted use, since it fulfils I_γ_ ≤ 6.

For the second chemical treatment, in which katoite and calcite were obtained as by-products, the following values were obtained: I_γ_ = 1.5 and I_γ_ = 1.4; and Ra_eq_ = 460 Bq/kg, Ra_eq_ = 430 Bq/kg. Both parameters exceeded the value demanded by current regulations, but were still 1.5 times lower than those obtained with the first chemical treatment, close to the value established by the regulations. This result was also to be expected, like the results for the previous by-products from the first chemical treatment, and the radionuclides present in starting residue (PG) were almost completely transferred to the katoite and to the final calcite sample. However, both by-products could be used to produce surface material with restricted use, since I_γ_ ≤ 6.

For the mortar sample, the value of I_γ_ = 0.8 and the Ra_eq_=250 Bq/kg were below I_γ_ = 1 and Ra_eq_ = 370 Bq/kg, respectively. Therefore, it can be stated that the mixture for the manufacture of mortar specimens produced with 1:3 by weight lime/sand and 0.50 water/lime ratios can be used practically without restriction to produce construction materials, due to the lower leaching results in comparison with those obtained in the PG sample.

## 4. Conclusions

### 4.1. PG

1. This waste does not pose any major leaching environmental risk, except for Ni.

### 4.2. Procedure A

2. Cd and Se concentrations in the different phases are moderately above the established limits. The other elements remained below the limits, except for U, Sr, and As. In the mortar, only Se, Cd, and U are above the legal limits.

3. The Ca containing by-products is NORM, but the mortar prepared with this series of lime was not.

4. The mobility of the different trace elements, both in the portlandite and calcite samples and in the mortars, were well below the established limit values, except for V.

### 4.3. Procedure B

5. The katoite contained amounts of Cd above the legally established value. Regarding the calcite, only Cd and U were above the limit permitted for uncontaminated soils. The resulting by-products are not NORM.

6. The metals in the katoite and calcite sample obtained turned out to be immobilized. Both by-products can be used for surface materials with restricted use.

## Figures and Tables

**Table 1:** Majority elements in both chemical processes.

Majority Elements (wt. %)	PG	C_S	PGAS	PGAB
Fe_2_O_3_	nd	nd	nd	0.01 ± 0.01
MnO	nd	nd	nd	nd
MgO	nd	nd	nd	nd
CaO	32 ± 1	49 ± 2	21 ± 1	32 ± 2
Na_2_O	0.01 ± 0.01	12.5 ± 0.6	20.5 ± 0.7	2.29 ± 0.08
K_2_O	0.02 ± 0.01	nd	0.02 ± 0.01	0.03 ± 0.01
TiO_2_	nd	nd	nd	nd
P_2_O_5_	0.65 ± 0.02	1.02 ± 0.01	0.38 ± 0.01	0.47 ± 0.01
SO_3_	46 ± 3	13.6 ± 0.2	27 ± 2	14.4 ± 0.9
Cl	nd	nd	nd	nd
F	nd	nd	nd	nd
SrO	nd	nd	nd	nd
BaO	nd	nd	nd	nd
LOI	18.4 ± 0.4	19.4 ± 0.2	14.4 ± 0.2	33 ± 0.1

wt. %: weight percentage; PG: phosphogypsum; C_S: lime putty; PGAS: katoite precipitate; PGAB: calcite from katoite carbonation.

**Table 2:** Contents of trace elements (mg/kg) in the PG (phosphogypsum) sample. Leaching results (mg/L) obtained from the application of the TCLP (Toxicity Characteristic Leaching Procedure) technique to the PG sample (L_f: phosphogypsum leaching), and reference (mg/kg) of the global average concentration of typical uncontaminated soils. The limit values permitted by the US-EPA (United States- Environmental Protection Agency) for metals in leachates extracted from the TCLP test and the maximum permissible limits of heavy metals in water for domestic use according to the US-EPA, WHO (World Health Organization), and EU (European Union) (according to German legislation).

Metal	PG (mg/kg)	L_f (mg/L)	Limits Allowed (mg/kg)	TCLP (U S—EPA)	U S—EPA	WHO	EU
V	2.9 ± 0.7	0.0161 ± 0.0001	97				0.05
Cr	6.3 ±0.2	< 0.023	92	5.0	0.10	0.05	0.05
Co	<0.6	<0.0024	17.3				
Ni	<3	0.026 ± 0.001	47			0.02	0.02
Zn	<42	0.600 ± 0.007	67		5.0	3.0	
As	<0.6	0.0052 ± 0.0004	4.8	5.0	0.05	0.01	0.01
Se	<30	<0.006	0.09	1.0			
Sr	360 ± 10	1.89 ± 0.03	320		4.0		
Cd	1.8 ± 0.4	0.0072 ± 0.0007	0.09	1.0	0.005	0.003	0.005
Ba	37 ± 1	0.0433 ± 0.0008	628	100.0	2.0	0.30	
Pb	1.8 ± 0.1	0.0053 ± 0.0001	17	5.0	0.015	0.01	0.01
Th	1.1 ± 0.2	<0.0013	10.5				
U	5 ± 1	0.0081 ± 0.0001	2.7				

**Table 3:** Trace elements of portlandite (C_S), calcite (Ca_S), and mortar samples. Leaching results obtained from the application of the TCLP (Toxicity Characteristic Leaching Procedure) technique to the sample of portlandite (L_p), calcite (L_c), and mortar (L_m).

METAL	C_S (mg/kg)	L_p (mg/L)	Ca_S (mg/kg)	L_c (mg/L)	Mortar (mg/kg)	L_m (mg/L)
V	<0.1	<0.007	<0.1	<0.007	0.3 ± 0.1	0.109 ± 0.001
Cr	13.8 ± 0.2	0.083 ± 0.003	11.5 ± 0.4	<0.023	9 ± 2	<0.023
Co	0.275 ± 0.004	<0.0024	0.23 ± 0.01	0.0048 ± 0.0002	0.165 ± 0.03	0.0049 ± 0.0002
Ni	1.80 ± 0.02	0.023 ± 0.001	2.3 ± 0.1	0.046 ± 0.001	0.347 ± 0.05	0.039 ± 0.001
Zn	10.1 ± 0.2	0.67 ± 0.02	9.7 ± 0.5	<0.214	9.6 ± 1.2	<0.221
As	7 ± 2	<0.002	5.2 ± 0.4	<0.002	3.9 ± 1.1	<0.002
Se	2.36 ± 0.08	<0.0059	2.33±0.08	<0.006	1.2 ± 0.7	<0.0061
Sr	413 ± 6	2.17 ± 0.05	353 ± 1	3.03 ± 0.04	170 ± 20	2.31 ± 0.01
Cd	3.37 ± 0.04	<0.0008	3.6 ± 0.1	<0.0008	1.4 ± 0.4	<0.0008
Ba	83 ± 2	0.062 ± 0.002	72 ± 1	0.1203 ± 0.0009	36 ± 2	0.0658 ± 0.0004
Pb	3.25 ± 0.05	0.0066 ± 0.0003	3.5 ± 0.2	<0.0009	1.43 ± 0.04	<0.0009
Th	1.5 ± 0.2	<0.0013	2.69 ± 0.08	<0.0013	0.32 ± 0.05	<0.0013
U	10.7 ± 0.3	<0.0012	9.7 ± 0.3	0.0508 ± 0.0003	3.9 ± 0.3	0.0302 ± 0.0003

**Table 4:** Contents of trace elements in the PGAS (katoite precipitate) and PGAB (calcite) samples. Leaching results obtained from the application of the TCLP (Toxicity Characteristic Leaching Procedure) technique to the PGAS (L_k) and PGAB (L_ck) samples.

Metal	PGAS (mg/kg)	L_k (mg/L)	PGAB (mg/kg)	L_ck (mg/L)
V	8.0 ± 0.3	<0.007	5.8 ± 0.3	0.0225 ± 0.04
Cr	5.3 ± 0.4	<0.023	6.7 ± 0.6	0.030 ± 0.004
Co	<0.6	<0.0024	<0.6	<0.0024
Ni	<3	0.0082 ± 0.0003	<3	0.0050 ± 0.0004
Zn	<41	0.0740 ± 0.0005	<41	<0.212
As	1.8 ± 0.8	<0.002	1.4 ± 0.2	<0.002
Se	<30	<0.006	<30	<0.0059
Sr	248 ± 10	1.69 ± 0.02	345 ± 5	1.46 ± 0.1
Cd	1.1 ± 0.2	<0.0008	1.6 ± 0.2	<0.0008
Ba	27 ± 3	0.0344 ± 0.0001	36 ± 1	0.0024 ± 0.02
Pb	1.5 ± 0.2	<0.0009	1.6 ± 0.1	<0.0009
Th	0.9 ± 0.1	<0.0013	1.1 ± 0.1	<0.0013
U	2.5 ± 0.3	<0.0012	2.9 ± 0.2	<0.0012

**Table 5:** Average activity concentrations of the different radionuclides present in chemical process phases. Average activity concentration with the TCLP (Toxicity Characteristic Leaching Procedure) leaching test of the different radionuclides and corresponding transfer factors (ŋ).

Sample	^226^Ra	^210^Pb	^40^K	^232^Th	^235^U
		Raw Material			
PG (Bq/kg)	(5.68 ± 0.25) 10^2^	(7.2 ± 0.3)10^2^	<111	4 ± 1	19 ± 3
TCLP (Bq/L)	0.65 ± 0.15 (ŋ = 2.3%)	4.0 ± 1.3(ŋ = 11.2%)	<3	<1	<2
PG + (Na(OH)					
Dissolution reaction					
C_S (Bq/kg)	(7.0 ± 0.3) 10^2^	(1.03 ± 0.05) 10^3^	16 ± 5	8 ± 1	28 ± 3
TCLP (Bq/L)	<1	<3	<3	<1	<2
Carbonation reaction					
Ca_S (Bq/kg)	(7.9 ± 0.3) 10^2^	(1.01 ± 0.07) 10^3^	<65	8 ± 1	20 ± 4
TCLP (Bq/L)	1.3 ± 0.4 (ŋ = 3.3%)	2.2 ± 1.1 (ŋ = 4.3%)	<2	<0.25	<0.2
Mortar production					
Mortar (Bq/kg)	(2.32 ± 0.10) 10^2^	(3.65 ± 0.17) 10^2^	80 ± 17	5.6 ± 0.5	8 ± 2
TCLP (Bq/L)	0.85 ± 0.25 (ŋ = 7.3%)	2 ± 1 (ŋ = 11.0%)	<2.5	<1	<1
PG + Caustic wastes.					
Dissolution reaction					
PGAS (Bq/kg)	(4.45 ± 0.19) 10^2^	(3.90 ± 0.18) 10^2^	<61	4 ± 1	10 ± 2
TCLP (Bq/L)	0.9 ± 0.2 (ŋ = 4.3%)	<3.5	<4	<1	<2
Carbonation reaction					
PGAB (Bq/kg)	(4.17 ± 0.17) 10^2^	(4.1 ± 0.3) 10^2^	<27	5 ± 1	10 ± 2
TCLP (Bq/L)	3 ± 1 (ŋ = 14.4%)	<3	<3	<1	<2
